# Modeling Neurovascular Disorders and Therapeutic Outcomes with Human-Induced Pluripotent Stem Cells

**DOI:** 10.3389/fbioe.2017.00087

**Published:** 2018-01-30

**Authors:** Allison M. Bosworth, Shannon L. Faley, Leon M. Bellan, Ethan S. Lippmann

**Affiliations:** ^1^Department of Biomedical Engineering, Vanderbilt University, Nashville, TN, United States; ^2^Department of Mechanical Engineering, Vanderbilt University, Nashville, TN, United States; ^3^Department of Chemical and Biomolecular Engineering, Vanderbilt University, Nashville, TN, United States

**Keywords:** induced pluripotent stem cell, blood–brain barrier, neurovascular unit, disease modeling, tissue engineering

## Abstract

The neurovascular unit (NVU) is composed of neurons, astrocytes, pericytes, and endothelial cells that form the blood–brain barrier (BBB). The NVU regulates material exchange between the bloodstream and the brain parenchyma, and its dysfunction is a primary or secondary cause of many cerebrovascular and neurodegenerative disorders. As such, there are substantial research thrusts in academia and industry toward building NVU models that mimic endogenous organization and function, which could be used to better understand disease mechanisms and assess drug efficacy. Human pluripotent stem cells, which can self-renew indefinitely and differentiate to almost any cell type in the body, are attractive for these models because they can provide a limitless source of individual cells from the NVU. In addition, human-induced pluripotent stem cells (iPSCs) offer the opportunity to build NVU models with an explicit genetic background and in the context of disease susceptibility. Herein, we review how iPSCs are being used to model neurovascular and neurodegenerative diseases, with particular focus on contributions of the BBB, and discuss existing technologies and emerging opportunities to merge these iPSC progenies with biomaterials platforms to create complex NVU systems that recreate the *in vivo* microenvironment.

## Introduction

The blood–brain barrier (BBB) maintains central nervous system (CNS) homeostasis by strictly regulating transport of ions, small molecules, proteins, and cells between the bloodstream and CNS (Obermeier et al., 2013). The BBB is formed by a monolayer of brain microvascular endothelial cells (BMECs), which express intercellular tight junctions that limit paracellular transport. Owing to the fidelity of these intercellular contacts, the BBB exhibits high transendothelial electrical resistance (TEER), a quantitative measure of barrier integrity performed by applying a voltage across the cell monolayer, measuring resulting current, and calculating resistance using Ohm’s Law. The BBB also expresses molecular transporters (e.g., GLUT-1, LAT-1) that shuttle nutrients and waste products and expresses efflux transporters that restrict the diffusion of lipophilic substances. These properties allow the BBB to protect the CNS neurons from harmful toxins and pathogens in the bloodstream, as well as regulate CNS homeostasis (Obermeier et al., 2013). Unfortunately, in aging and the progression of various disease states, such as Alzheimer’s disease (AD), multiple sclerosis, and traumatic brain injury, many of these BBB-specific properties are disrupted or lost (Korn et al., 2005; Marques et al., 2013; Friese et al., 2014). As such, improved understanding of BBB function and its alterations in disease may lead to new strategies for therapeutic intervention in neurological and neurodegenerative disorders.

*In vitro* models of the BBB are useful for understanding proper endothelial cell functionality and gaining insight into disease mechanisms. Primary mouse, rat, bovine, and porcine BBB endothelium have been often used for constructing various *in vitro* models (Helms et al., 2016), but it has been recognized that non-human cell sources are often insufficient for modeling human mechanisms because of species’ differences in receptor and transporter expression levels and homology (Syvänen et al., 2009; Helms et al., 2016). Therefore, human cell sources would be preferred in many cases. However, primary human BBB endothelial cells exhibit only moderate barrier functionality *in vitro* are usually very difficult and time consuming to isolate and can only be obtained in low yield (approximately 1 million cells per 5–10 mm^3^ tissue) (Bernas et al., 2010). Patient heterogeneity also provides an additional obstacle to the reproducibility of primary human cells. Immortalized BBB endothelial cell lines have been tested as an alternative to primary cells because they bypass the process of isolation from tissue and are derived from a clonal source (Weksler et al., 2005), but the immortalization process typically yields poor barrier functionality.

**Figure 1:**
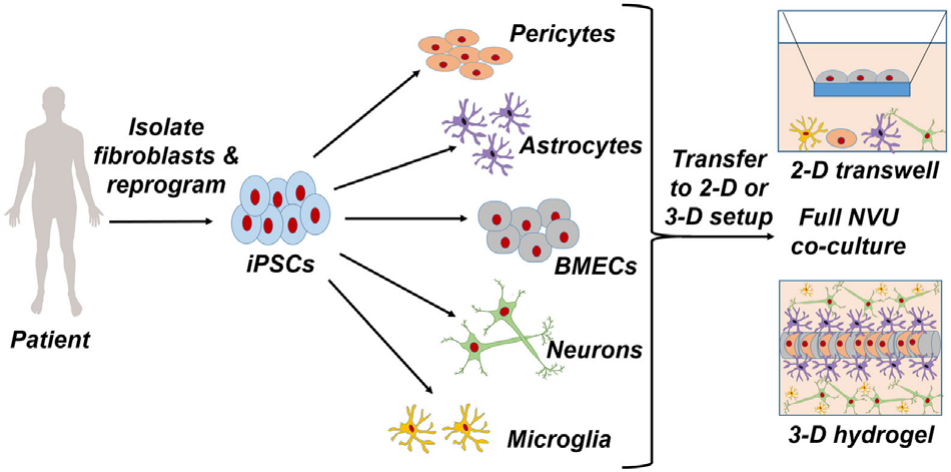
Patient-specific fibroblasts are isolated and reprogrammed to a pluripotent state, from which pericytes, astrocytes, brain microvascular endothelial cells, neurons, and microglia are differentiated. Full coculture models of the neurovascular unit are achieved through a two-dimensional Transwell setup or a three-dimensional hydrogel setup.

These issues with primary cells and immortalized cell lines have led to the exploration of human-induced pluripotent stem cells (iPSCs) as a new cell source for modeling BBB. iPSCs are characterized by their ability to proliferate indefinitely and differentiate into any cell type of the human body (Takahashi et al., 2007; Yu et al., 2007). Recent work has shown that iPSCs can be differentiated into BBB endothelial cells (Lippmann et al., 2012), and follow-up studies have improved on this differentiation process to produce cells that have properties approaching *in vivo* characteristics (Lippmann et al., 2014). In addition, the presence of astrocytes and pericytes, which reside in the neurovascular unit (NVU) and further support BBB function *in vivo*, can similarly enhance the BBB phenotype *in vitro* (Hollmann et al., 2017). Finally, the use of biomaterials such as hydrogels has facilitated the development of three-dimensional models that can prospectively mimic NVU architecture. Figure 1 illustrates the general process by which patient-derived cells can be incorporated into such models. In this review, we summarize these advancements in BBB modeling with iPSCs, discuss how iPSC-derived BBB endothelium could be used to enhance neurodegenerative disease mechanistic interrogations and drug screening campaigns, and outline engineering and fabrication approaches that may be used in future studies to produce NVU models with more predictive power.

## Modeling the BBB with iPSCs

As described above, the BBB is characterized by specialized properties such as high TEER, low passive permeability to both hydrophilic and hydrophobic substances, and expression of a bevy of molecular transporters. The differentiation of iPSCs to endothelial cells with these properties was first described in the seminal work (Lippmann et al., 2012). This procedure begins with a codifferentiation process that generates both neural and endothelial cells to mimic endogenous neurovascular development, whereby neural progenitors impart BBB identity onto endothelial cells. Wnt/β-catenin signaling plays a key role in this process *in vivo* (Stenman et al., 2008; Daneman et al., 2009) and was therefore assayed in the iPSC system; it was determined that several key WNTs were expressed during differentiation, and localization of β-catenin to the nucleus of PECAM-1^+^ endothelial cells was found to increase throughout the differentiation process, indicating that Wnt/β-catenin signaling was activated. Endothelial cells were then purified from the heterogeneous mixture by selective adhesion to collagen IV and fibronectin. Expression of endothelial and tight junction markers, as well as active efflux transporter expression and representative permeability to a panel of small molecules, confirmed BBB-like identity. Finally, TEER was measured at ~800 Ω × cm^2^ after coculture with astrocytes; although this level of barrier fidelity was higher than any previous human model, it still decreased well below measured values in rats (up to 5,900 Ω × cm^2^) (Butt et al., 1990) and theoretical maximums calculated by radioactive ion permeability (~8,000 Ω × cm^2^) (Smith and Rapoport, 1986).

Animal and human models of the developing brain have shown that radial glial cell-secreted retinoic acid (RA) helps induce BBB properties (Mizee et al., 2013). For this reason, follow-up work to optimize BMEC differentiation methods used media supplemented with RA to boost barrier properties in iPSC-derived BBB endothelial cells. RA treatment during the differentiation process yielded cells with increased expression of VE-cadherin and occludin and drastically elevated TEER values to ~3,000 Ω × cm^2^ (Lippmann et al., 2014). Further optimization of seeding density (Wilson et al., 2015) and defined medium composition (Hollmann et al., 2017) have shortened the differentiation time from 13 to 8 days, with the final BBB population still exhibiting excellent barrier properties (TEER > 6,000 Ω × cm^2^ upon coculture with astrocytes and pericytes). The reproducibility of the differentiation procedure has been subsequently confirmed in several publications all demonstrating BBB identity through elevated TEER and other molecular characterizations (Katt et al., 2016; Appelt-Menzel et al., 2017; Kokubu et al., 2017; Lim et al., 2017; Wang et al., 2017b). While initial protocols yielded stable TEER measurements (≥1,000 Ω × cm^2^) for approximately 4 days (Lippmann et al., 2014), more recent protocols have yielded stable measurements for up to 14 days (Hollmann et al., 2017). As these protocols advance and more closely resemble *in vivo* conditions, it is expected that TEER measurements will stabilize even further and extend over longer periods of time.

The availability of these high-quality human BMECs has spurred numerous advancements in disease modeling. Other than their excellent barrier properties relative to other sources of human BMECs (Helms et al., 2016), the core utility of iPSC-derived BMECs is their derivation from a specific genetic background, which permits explicit studies of genotype/phenotype linkages. This powerful approach was applied in two recent studies. First, iPSCs from patients with Allan–Herndon–Dudley syndrome (AHDS; characterized by severe neuropsychomotor impairments) were used to study BBB transport properties (Vatine et al., 2017). AHDS is caused by inactivating mutations in monocarboxylate transporter 8, which is a thyroid hormone transporter, but the mechanism of the disease has been unclear. Although iPSC-derived neurons developed normally in the presence of thyroid hormone, BBB endothelial cells derived from AHDS iPSCs were deficient at transporting thyroid hormone in an apical-to-basolateral direction (“blood” to “brain”). These results imply that AHDS could potentially be corrected if this delivery problem were overcome. Moreover, this study in iPSCs was particularly crucial because rodents express a separate transporter (Oatp1c1) at the BBB that can transport thyroid hormone, which is absent at the human BBB; thus, to accurately mimic the disease, it was particularly crucial to work with human cells. The second study to explore the contribution of human genetics to BBB disease utilized Huntington’s disease (HD) iPSCs (Lim et al., 2017). BBB deficits have been observed in live measurements in HD patients and within postmortem tissue analyses, but it was unclear if these phenotypes were cell autonomous or due to secondary damage incurred by neural inflammation and death. Thus, using the standard iPSC-to-BMEC differentiation procedure (Lippmann et al., 2014), it was observed that BMECs differentiated from HD-iPSCs had defects in barrier formation, including diminished TEER and increased transcytosis. Further analyses suggested that these malfunctioning barrier properties may be related to an increased proliferative/angiogenic capacity. Intriguingly, the severity of most BBB defects increased with the number of CAG repeats in the huntingtin protein, which also correlates with the severity of the disease *in vivo*. These results suggest that defects in the BBB could potentially contribute to HD onset and progression.

In the future, we hypothesize that other diseases with explicit changes or deficits in BBB function will be modeled with iPSCs. For example, cerebral cavernous malformations (CCMs), which are vascular malformations found predominantly in the CNS that cause hemorrhagic stroke, are caused by the loss of function mutations in three genes that form an intracellular adaptor protein complex (Cavalcanti et al., 2011). However, the clinical course of the familial form of the disease is highly variable, suggesting additional genetic modifiers could play a role in disease progression. For example, a recent study demonstrated that polymorphisms in the innate immunity gene *TLR4* are associated with higher disease burden in humans (Tang et al., 2017a). iPSCs from CCM patients could be a powerful tool for interrogating the genetic and environmental factors that exacerbate this disease. Another disease that may benefit from iPSC modeling is drug-resistant epilepsy. Nearly one-third of epilepsy patients are refractory to pharmacotherapy, and the mechanism of this drug resistance is hotly debated (Tang et al., 2017b). Much of the clinical data in drug-resistant epilepsy has focused on polymorphisms and expression changes in efflux transporters at the BBB. iPSCs would again represent an excellent tool for studying the influence of transporter gene polymorphisms on protein expression levels and drug pharmacology. Overall, iPSCs do not even need to be derived from specific patient populations to be useful for these applications. Several studies have assayed the responses of iPSC-derived BMECs to hypoxia and glucose deprivation in an effort to mimic ischemia (Page et al., 2016; Kokubu et al., 2017), and these types of mechanistic analyses into basic BBB function under disease-like conditions can be conducted with generic iPSCs. Moreover, cutting-edged genome engineering techniques [e.g. CRISPR/Cas (Cong et al., 2013; Mali et al., 2013)] now allow researchers to remove genes or introduce specific mutations into iPSCs (González et al., 2014; Mandegar et al., 2016). As such, the ability to make isogenic pairs of iPSCs will most likely be leveraged in the future to shed light on the genetics of BBB dysfunction.

## How iPSC-Derived BBB Endothelium Can Provide Insight into Neurovascular Disease Mechanisms and Treatment Strategies

In the previous section, we described current and theoretical examples for how iPSCs can be used to study diseases that directly influence BBB function. However, BBB dysfunction has been observed in many neurodegenerative diseases, including AD, Parkinson’s disease (PD), and amyotrophic lateral sclerosis (ALS); whether this dysfunction causes the degeneration or is secondary to the diseases and simply exacerbates their progression remains to be determined. iPSCs represent a potential route for deconstructing neurovascular changes in these disorders, especially given the progress in differentiating other NVU cell types including neurons, microglia, pericytes, and astrocytes (Chambers et al., 2009; Orlova et al., 2014; Chandrasekaran et al., 2016; Pandya et al., 2017). Below we describe some of these disorders and current routes for modeling them with iPSCs, as well as advancements that could be realized by incorporating iPSC-derived BBB endothelium into existing model systems. This list is by no means all encompassing and is intended to mainly provide the readers with intriguing research avenues and questions.

### Alzheimer’s Disease

Hallmarks of AD pathology include the deposition of amyloid-β (A-β) in the extracellular space and buildup of hyperphosphorylated tau fibers in the cytoplasm of neurons (Selkoe, 2001). Genetic mutations in the β-amyloid precursor protein (APP) and presenilin genes have been linked to cases of familial AD. Israel et al. (2012) were the first to use iPSC-derived neurons from patients with APP genetic duplications and patients with sporadic AD to assess disease mechanisms *in vitro*. They reported that the APP genetic mutation led to increased levels of pathological markers A-β and phosphor-tau. Other studies have shown that familial AD-derived iPSCs produce neurons with altered Aβ42/Aβ40 ratios (Mertens et al., 2013; Muratore et al., 2014), which recapitulates a key phenotype observed in the CSF of AD patients (Borchelt et al., 1996; Kumar-Singh et al., 2006). Given these findings, how then could iPSC-derived BMECs be incorporated with these defective neurons and other neurovascular cell types to model disease progression? For one, both soluble Aβ1–40 (Hartz et al., 2016) and Aβ1–42 (Park et al., 2014) have been shown to reduce p-glycoprotein expression at the BBB in animal models; as such, loss of p-glycoprotein in human neurovascular models may be a point of interest. Second, human ApoE4, but not ApoE2 or ApoE3, has been implicated in neurovascular breakdown (Bell et al., 2012); ApoE4 is a major genetic risk factor in AD, and iPSC-derived neurovascular models represent an excellent system to study its effects in a human genetic background. Third, PICALM, another genetic risk factor for AD, is expressed at the BBB and involved in amyloid clearance *via* transcytosis; iPSC-derived endothelial cells (albeit not BBB-specific) carrying a protective PICALM allele exhibit increased amyloid transport (Zhao et al., 2015). Ostensibly, the regulation of PICALM expression and prospective drugs that increase its expression or activity could be screened in iPSC-derived brain endothelium. More broadly, because amyloid clearance occurs at least in part through the vasculature and is implicated in AD progression (Sagare et al., 2012), its incorporation into human neurovascular models is vital for understanding plaque and tangle accumulation and removal.

### Parkinson’s Disease

Parkinson’s disease features the main clinical symptom of bradykinesia resulting from the loss of dopaminergic neurons in the substantia nigra (Lees et al., 2009). Familial versions of this disease can be traced to mutations in the Parkin, LRRK2, and α-synuclein proteins, which lead to phenotypes such as compromised mitochondrial functionality and aggregation of α-synuclein to form Lewy bodies. iPSCs derived from patients with Parkin mutations can recapitulate PD phenotype *in vitro* (Imaizumi et al., 2012), including impaired mitochondrial function, accumulation of α-synuclein in differentiated neurons, and formation of Lewy bodies that corresponded to the structures found in the patient’s postmortem brain tissue. Meanwhile, neurons derived from the iPSCs of patients with LRRK2 mutations are more susceptible to stress activators, leading to caspase activation and cell death (Nguyen et al., 2011). Neurons derived from the iPSCs of patients with triplication of the *SNCA* gene (which encodes α-synuclein) exhibit elevated α-synuclein protein expression, thus recapitulating the *in vivo* phenotype (Devine et al., 2011). More recently, iPSC-derived neurons from patients with a different *SNCA* mutation were shown to exhibit protein aggregation and fragment axons, which could be rescued through small molecule treatment (Kouroupi et al., 2017). Intriguingly, preformed α-synuclein fibrils can influence tight junction expression in a human *in vitro* BBB model (Kuan et al., 2016); however, the model used in this case (hCMEC/D3 immortalized cells) is notably not very tight (TEER less than 20 Ω × cm^2^). Given the more significant tightness of iPSC-derived BMECs, accumulation and/or transport of α-synuclein, and its influence on barrier function, could be examined in a more physiologically relevant model system. This is particularly relevant given recent findings that α-synuclein assemblies can cross the BBB *in vivo* (Sui et al., 2014; Peelaerts et al., 2015) and that iPSC-derived BMECs derived from a patient with *PARK2* mutations may have defective or altered p-glycoprotein expression (Hollmann et al., 2017).

### Amyotrophic Lateral Sclerosis

Amyotrophic lateral sclerosis is characterized by axonal degeneration and ultimately death of the neuronal cell body in CNS motor neurons. While it has been shown that this neurodegeneration is correlated with protein accumulation in motor neurons (Hirano et al., 1984), the underlying mechanisms of protein accumulation and how it leads to selective degradation of motor neurons is still largely unknown. To study this disease using iPSCs, many have chosen to look at the superoxide dismutase (SOD1) gene mutation since it is responsible for approximately 20% of all cases of familial ALS (Sau et al., 2007). Although familial cases account for only 5–10% of total ALS cases, many phenotypic similarities occur among sporadic and familial types, so it is still viewed as informative to study the disease using SOD1 mutations. Chen et al. (2014) used this approach to assess neurofilament aggregation and neurite degeneration in iPSC-derived motor neurons containing the SOD1 gene. They found that mutant SOD1 binds the mRNA of NF-L, a neurofilament subunit, leading to an overall reduction in NF-L levels and altered neurofilament subunit ratios in ALS motor neurons. This was predicted to be an early event in ALS onset, later leading to neurite degeneration. Meanwhile, Kiskinis et al. (2014) used isogenic pairs of iPSCs (wild-type and mutant SOD1) to show that the SOD1 mutation alters a variety of transcriptional signatures in motor neurons, including upregulation of stress pathways. Others have used iPSC-derived motor neurons to reveal a hyperexcitability phenotype that is broadly applicable to many ALS gene mutations, including variants of SOD1, C9ORF72, and TARDBP (Wainger et al., 2014; Devlin et al., 2015). These particular findings have led to an ALS clinical trial using ezogabine, an approved antiepileptic and Kv7.2/3 potassium channel agonist (McNeish et al., 2015), which presumably crosses the BBB in rodents (Large et al., 2012). Ideally, the permeation of this compound through the human BBB, as well as other variants and classes of potassium channel agonists, could be tested within the iPSC model. However, correcting aberrant phenotypes solely in motor neurons may not cure ALS, as mutant astrocytes are also selectively toxic to motor neurons (Di Giorgio et al., 2007, 2008; Nagai et al., 2007; Marchetto et al., 2008). It is intriguing to further note that vascular disruption has been noted in ALS using cell culture models (Meister et al., 2015), animal models (Zhong et al., 2008; Winkler et al., 2014), and postmortem human tissue (Garbuzova-Davis et al., 2012; Winkler et al., 2013). Of particular interest, in the animal models, BBB disruption precedes motor neuron death. Whether this vascular degeneration is a direct cause of the ALS-causing mutations or due to altered crosstalk with astrocytes (which clearly have toxic capacity) or another NVU cell type remains to be determined, but the iPSC model represents a possible route for deconstructing these disease mechanisms and ultimately elucidating the role of BBB dysfunction in disease progression.

In addition to improving the accuracy of disease modeling applications, inclusion of a BBB component in these models also facilitates a more accurate prediction of drug outcomes (as alluded to in reference to the ezogabine clinical trial). Academia has made significant strides in screening both existing and newly developed drugs within iPSC-derived neurological models (Avior et al., 2016), including models of complex behavioral disorders such as autism (Shcheglovitov et al., 2013; Griesi-Oliveira et al., 2015; Mariani et al., 2015; Forrest et al., 2017) and schizophrenia (Brennand et al., 2011; Hook et al., 2014; Wen et al., 2014; Yoon et al., 2014). These models report drug efficacy among human diseased and control lines and provide perspectives on how they may be implemented clinically. However, as mentioned previously, the BBB blocks transport of ions, small molecules, and proteins between the bloodstream and the CNS. As such, an estimated 98% of all small molecules do not cross the BBB in appreciable amounts (Pardridge, 2005). Therefore, if a drug that exhibits effectiveness against neural cultures is not BBB penetrant, it will likely be ineffective in treating diseased CNS tissue, which represents a major constraint within the drug development process. Given the overall difficulties in CNS drug discovery (Choi et al., 2014), we believe that the addition of a BBB component is vital toward modeling complex CNS disorders and accurately predicting drug delivery and responses.

## Routes for Engineering Complex *In Vitro* Neurovascular Models Using iPSCs

Much of the discussion in the above section focused on the prospective interplay between BMECs and other resident cell types in the NVU. Historically, such crosstalk has been explored primarily in planar cultures or Transwell setups. However, 2D cell culture platforms overall are often inadequate as model tissue systems due to their inability to support truly biomimetic cell–cell and cell–matrix interactions and thus are unable to fully integrate the complex biochemical and mechanical cues affecting cell homeostasis and responses to environmental perturbations (Pampaloni et al., 2007; Edmondson et al., 2014; Banerjee et al., 2016; Helms et al., 2016). For this reason, there is a need to adopt 3D models that better recapitulate the native cellular environment to achieve *in vitro* model systems capable of yielding accurate predictions regarding factors that influence both disease progression and useful clinical interventions. This need has driven efforts in biomaterials patterning and microfluidic fabrication methods that enable the production of 3D cell culture systems with cellular constituents adopting behavior that more closely mimics that observed *in vivo* (Huh et al., 2011; Wikswo, 2014; Ravi et al., 2015). For the purposes of this review, we discuss a range of techniques that could be used to derive 3D neurovascular models, many of which were initially validated using non-stem cell sources, but nonetheless reflect significant advances in tissue engineering with the potential to provide insights into BBB and NVU function not currently obtainable in 2D formats (Cucullo et al., 2008). To develop truly biomimetic tissue models, however, researchers must still overcome several challenges, one of which is the need to develop approaches that incorporate iPSC-derived cells in these complex platforms.

Microfluidic fabrication techniques provide a powerful method for constructing NVU models in a highly controlled, perfused environment. Microfluidic BBB models, some commercially available (Prabhakarpandian et al., 2013; Lamberti et al., 2014), have proven useful for examining the impact of shear stress and scaffold geometry on brain endothelium function and morphology, as well as for drug screening applications (Cucullo et al., 2008; Booth and Kim, 2012; Yeon et al., 2012; Griep et al., 2013; Ye et al., 2014; Sellgren et al., 2015). While most of these platforms were developed using non-stem cell sources, a recent report of iPSC-derived BMECs cocultured with astrocytes on opposite sides of a porous membrane housed within a microfluidic channel indicated that these cells maintained a robust, *in vivo*-like barrier in the presence of continuous fluid flow for up to 12 days (Wang et al., 2017b).

**Figure 2:**
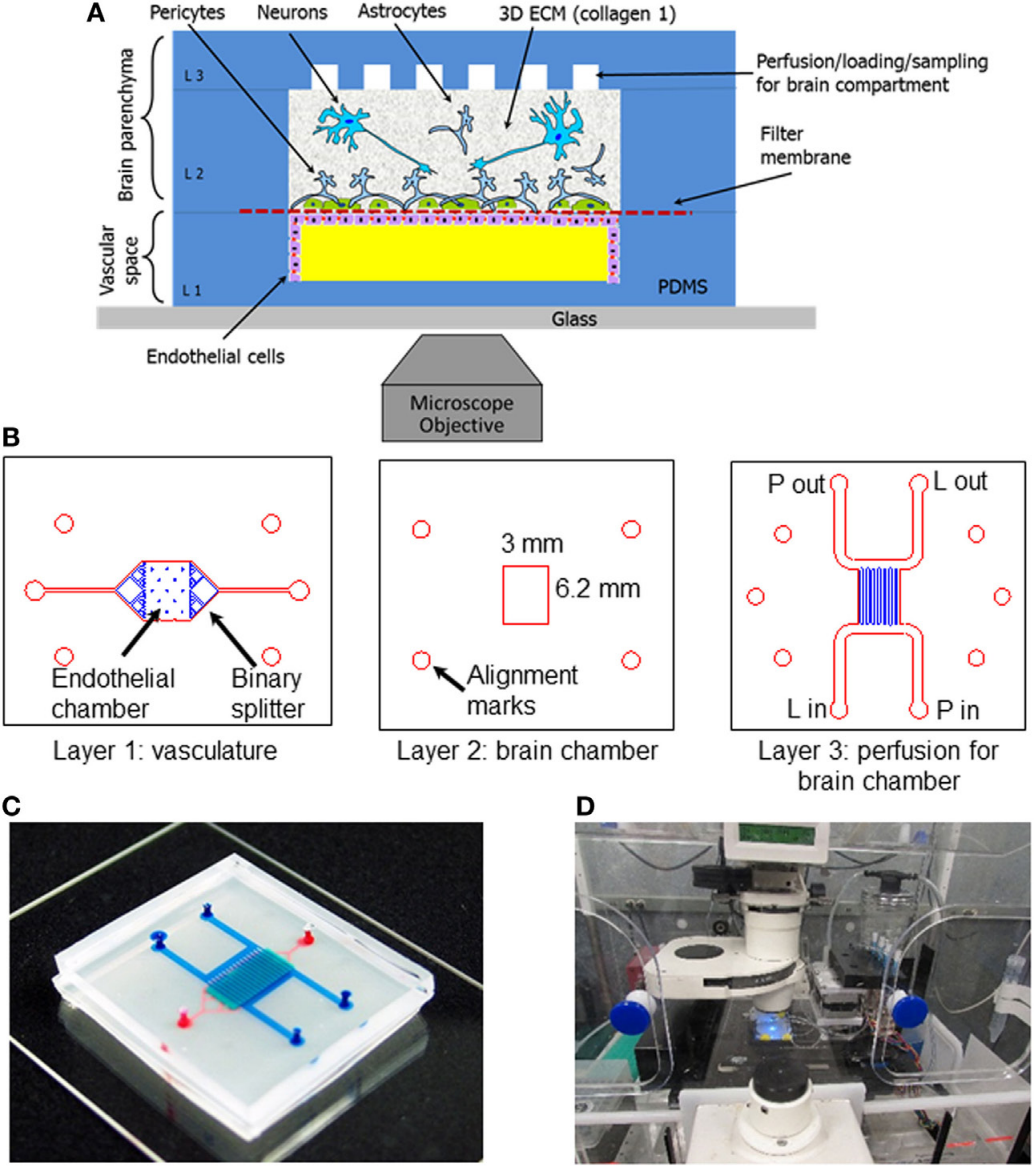
Schematic and experimental setup of the neurovascular unit (NVU)-on-a-chip. **(A)** Schematic view of the neurovascular unit (NVU) indicating major components, cell types, and their spatial arrangement. **(B)** Photolithographic masks used to fabricate the three-layered NVU. **(C)** A photograph of the assembled NVU loaded with colored dyes indicating different compartments: red = vasculature; semi-transparent white = filter membrane; turquoise = brain compartment; and blue = brain perfusion. **(D)** NVU device on an incubated microscope stage. Reproduced from Brown et al. (2015), with the permission of AIP Publishing.

Microfluidic BBB models are particularly well-suited to high-throughput, massively parallel drug screening efforts. Typically, microfluidic platforms are based on polydimethylsiloxane (PDMS) or glass substrates, which, while conducive to long-term cell culture, fail to recapitulate the complex 3D microenvironment of natural tissue. Scaffolds fabricated from hydrogel matrices are appealing for modeling the NVU, in that they offer a more physiologically representative platform in terms of stiffness, architecture, degradability, and a means by which to allow more natural interactions with surrounding cell populations (Tibbitt and Anseth, 2009). Hybrid platforms have emerged that incorporate hydrogel-filled channels or compartments to provide tissue-specific biological cues within a microfluidic format. This approach facilitates the use of fragile hydrogels composed of natural matrix proteins such as collagen, fibronectin, and hyaluronic acid that, depending on the concentration, often lack the structural integrity to support integrated fluidic channels in 3D as a free-standing unit. Such microfluidic compartments, filled with hydrogels containing endothelial cells, have been shown to be conducive to “bottom-up” formation of vascularized constructs through cell-driven angiogenic processes (Phan et al., 2017; Wang et al., 2017a). These methods have also been used in highly complex, organ-on-a-chip platforms. Composed of modular components of cells grown in hydrogel matrices as well as those cultured on porous membranes connected by microfluidic channels, organ-on-a-chip systems provide a potentially powerful method for gaining critical insights into population-specific responses to environmental perturbations with multiple readout mechanisms (Markov et al., 2012; Brown et al., 2015; Herland et al., 2016; Adriani et al., 2017). As illustrated by the experimental setup in Figure 2, the compartmentalized aspect of organ-on-a-chip systems provide a novel mechanism for intrapopulation and interpopulation soluble communication that is incredibly useful in determining specific response profiles of individual cell population to toxic/therapeutic compounds in combination with downstream impacts on neighboring cell compartments. As such, these platforms are ideal for analyzing both drug permeability and drug metabolism for pharmacokinetic and pharmacodynamic modeling. However, scaffolds designed to identify critical biological mechanisms underlying pathological conditions should ideally be more biomimetic, such that the scale, biological matrix, cellular components, and organization better approximate physiological processes, including both soluble and contact-based cellular interactions. Furthermore, none of these platforms have yet to incorporate matched cells derived from stem cell sources, which would further enhance the ability to represent native physiological systems.

Microfluidic fabrication methods often require highly specialized equipment and training that are typically outside the scope of standard biological laboratory facilities and staff experience. In contrast, incorporation of hydrogel matrices into standard culture platforms is generally simpler and more scalable; although microfluidic NVU devices are generally fabricated to be less than 1 mm in thickness, hydrogel platforms are only limited by the size of the culture vessel as long as the construct can be appropriately perfused. 3D NVU models created based on the hydrogel scaffolds have thus far yielded promising results. Cells cultured on the surface of chemically defined synthetic PEG hydrogels have been shown to self-assemble into biomimetic NVU constructs have been used for high-throughput toxicity screening (Murphy et al., 2010; Pellett et al., 2015; Schwartz et al., 2015; Zanotelli et al., 2016; Barry et al., 2017). Others have shown that embedding neural cells in alginate hydrogels promotes self-assembly of “BBB spheroids,” composed of an astrocytic core sheathed in layers of pericytes and brain endothelial cells, which may be useful for drug screening applications (Nguyen et al., 2013; Cho et al., 2017). Non-differentiated cells encapsulated in hydrogel matrices and directed through combined mechanical cues and growth factors offer an attractive method for yielding complex multicellular constructs that mimic *in vivo* cellular organization (Bozza et al., 2014; Cho et al., 2017). However, these all lack the integrated, perfused vasculature necessary for a truly biomimetic model.

Like the aforementioned PDMS microfluidic platforms, hydrogels can also be patterned as free-standing constructs with perfusable internal channel networks (Faley et al., 2015). In fact, initial efforts in generating vascular models in biomimetic hydrogels scaffolds utilized lithographic and soft templating techniques adopted from traditional microfluidic fabrication methods (Cabodi et al., 2005; Golden and Tien, 2007; Zheng et al., 2012). These approaches are extremely good at producing micron and submicron patterns, but scaling the resulting devices to achieve tissue-scale constructs often requires cumbersome sequential assembly that is not particularly well suited to cell-laden scaffolds. Recent advances in 3D printing allow large-scale patterning with cell-laden bioinks in combination with sacrificial templates to yield integrated channel networks (Miller et al., 2012; Bertassoni et al., 2014; Kolesky et al., 2014). Currently, however, printer resolution limitations generally make fabricating channels in 3D printed scaffolds below 100 μm in diameter impractical. Still, these platforms allow modeling of arteriole (and larger) sized structures in an immediately perfusable format that is easily tailored to accommodate variations in shear, mechanical forces, cellular organization, and soluble signaling factors, thereby mimicking natural tissue in structure and scale. Only a few studies have used these techniques to examine brain endothelial dynamics using differentiated stem cells in fluidic hydrogel scaffolds (Jiménez-Torres et al., 2016; Ingram et al., 2017), but none have yielded endothelial lumens with appreciable barrier strength.

The next step in the fabrication of a biomimetic *in vitro* neurovascular model involves integrating the latest advances in iPSC derivation methods along with tissue engineering approaches for generating capillary-sized vascular structures. As opposed to extrusion-based 3D printing, projection stereolithographic printing techniques can produce 3D scaffolds with complex integrated fluidic channels (diameters as small as 20 μm) through spatially controlled cross-linkage of photosensitive prepolymer solution by targeted light exposure, delivered by computer-controlled digital micro-mirror assemblies or through a 2D “mask” in a layer-by layer fashion (Hribar et al., 2015; Wang et al., 2015; Raman et al., 2016; Valentin et al., 2017). Laser-assisted printing operates in a similar fashion, except that the laser focus is traditionally “patterned” by CAD file and, in the case of two-photon systems utilizing pulsed femtosecond laser sources, has achieved pattering of hydrogel features as small as 10 μm (Hoffmann and West, 2013). In addition, a novel hybrid approach recently demonstrated the ability to generate 20-μm channels using multiphoton stereolithography to photo-bleach biotin-4-fluorescein in unpolymerized collagen (Skylar-Scott et al., 2016). This particular method is capable of achieving down to 1-μm resolution for patterning much smaller structures, but at the expense of scalability with overall scaffold thicknesses limited to 1 mm. While lithographic methods such as these allow for reproducible fabrication of complex fluidic architecture of capillary scale within hydrogel scaffolds, the toxic and/or mutagenic effects of high-intensity laser exposure, photoinitiating components, and radicals/harmful byproducts produced during fabrication remain a significant concern, especially when applied to highly sensitive stem cell-derived populations. Laser ablation lithography attempts to circumvent the concerns associated with photo-initiated hydrogel polymerization by sculpting channels in blocks of hydrogels after polymerization by more cell-friendly processes (Brandenberg and Lutolf, 2016). However, the specialized equipment and necessary skills to implement most of these lithographic fabrication methods comprise the greatest obstacle to becoming a technique widely accessible to the broader neurovascular modeling community.

In contrast, a technologically straightforward approach to fabricating capillary-sized channels is to simply embed a sacrificial mesh of microscale fibers within the hydrogel matrix that, after polymerization, is washed away. The utility of this approach for developing biomimetic tissue constructs is entirely contingent upon identifying a sacrificial material that is non-cytotoxic, easily spun into microfibers, insoluble in water at 37°C, and can be removed from the crosslinked hydrogel using non-cytotoxic methods. A recent report demonstrating the ability to generate capillary-like channels from thermoresponsive poly-NIPAM fibers. The unique LCST properties of P-NIPAM result in shift from water-insoluble to water-soluble when temperatures fall below 32°C, enabling facile fiber removal at room temperature yielding immediately perfusable microscale channels that are promising for modeling brain microvasculature networks (Lee et al., 2016).

Other than fabrication, another hurdle for modeling complex neurovascular assemblies is the incompatibility of a traditional biological readout for validating the integrity and the functionality of brain endothelium. Measuring the resistance of the endothelial barrier (TEER) is the most common method for assessing barrier strength in 2D cultures, but obtaining reliable resistance measurements from endothelial lumens lining perfused channel networks within 3D hydrogel scaffolds is not realistic (Srinivasan et al., 2015). For this reason, the permeability of endothelial layers in hydrogel channels is typically evaluated by monitoring tracer diffusion across cell barriers using compound such as radiolabeled or fluorescently conjugated compounds (Bowman et al., 1983; Franke et al., 1999; Lippmann et al., 2013; Hollmann et al., 2017). Calculating the permeability coefficients from these observations is straightforward for non-intersecting channels in 2D arrays (Zheng et al., 2012). However, randomly intersecting channels in 3D matrices that mimic physiological architecture introduce a significant degree of complexity for establishing quantitative values of lumen permeability. Furthermore, tracers can perturb cellular activity and consequently effect barrier integrity. Thus, alternative methods, ideally those that are non-invasive and allow continuous monitoring of barrier integrity, are needed to realize the full potential of these newly emerging biomimetic neurovascular models. One solution may be to leverage advances in CRISPR technologies to produce iPSC lines that include reporters of cell function, including barrier integrity. Overall, recent innovations in 3D cell scaffold fabrication techniques, iPSC derivation methods, and genome editing have facilitated this exciting juncture in the field of tissue engineering; these progressive resources should ultimately facilitate the development of complex, truly biomimetic *in vitro* models of the NVU.

## Discussion

Major strides have been made toward building BBB models that take advantage of human iPSC technology. In addition, ever-better microfluidics platforms and perfusable hydrogels are being developed to provide three-dimensional architectures that better mimic *in vivo* vessel structures. Valuable insights into neurological diseases have already been reported using iPSC-based model systems, and it is expected that these models will improve further when combined with novel biomaterial scaffolds into full NVU constructs. Once built, these complex *in vitro* models are poised to provide relevant clinical knowledge regarding debilitating cerebrovascular diseases and ultimately facilitate the next generation of therapeutic interventions.

## Author Contributions

All authors contributed to the preparation of this review.

## Conflict of Interest Statement

The authors declare that the research was conducted in the absence of any commercial or financial relationships that could be construed as a potential conflict of interest.
